# Comparison of the Effect of SAMPE and ALDERTE Checklists on the Incidence of Complications After Discharge of Radical Prostatectomy Patients from the Post-anesthesia Care Unit (PACU)

**DOI:** 10.5812/aapm-156738

**Published:** 2025-01-15

**Authors:** Alireza Zareie, Parisa Moradimajd, Azam Saei, Jamileh Abolghasemi

**Affiliations:** 1 Department of Anesthesia, Allied Medical School, Iran University of Medical Sciences, Tehran, Iran; 2 Research Center for Trauma in Police Operations, Directorate of Health, Rescue & Treatment, Police Headquarter, Tehran, Iran

**Keywords:** Anesthesia, Checklist, Prostatectomy, Patient Discharge, Recovery Room

## Abstract

**Background:**

Among the available tools, the SAMPE and ALDERTE checklists have been specifically designed to facilitate timely patient discharge, minimize human error, and optimize resource utilization. Given the complexities associated with surgical care, a comparative analysis of these two checklists is essential to evaluate their efficacy in improving discharge outcomes and preventing complications.

**Methods:**

This descriptive-analytical cross-sectional study assessed the distribution of complications following radical prostatectomy surgery by utilizing the SAMPE and ALDERTE checklists for discharge from the post-anesthesia care unit (PACU). A total of 156 participants, divided into three groups of 52 individuals each, were monitored for post-discharge complications 12 hours after their discharge from the PACU across three training centers. This methodology enabled a thorough evaluation of the roles of both checklists in mitigating adverse events during the critical post-operative period.

**Results:**

No significant differences in complication rates were observed among the groups; however, bleeding and vomiting were slightly more common in the SAMPE group.

**Conclusions:**

This study concluded that neither the SAMPE nor the ALDERTE checklist provided a distinct advantage over the control group, which comprised patients routinely discharged from the same treatment center. Both checklists demonstrated similar functionalities, with each showing relative strengths in specific aspects; however, neither was found to be universally superior to the other.

## 1. Background

Prostate cancer ranks as the second most prevalent malignancy after skin cancer and is the second leading cause of cancer-related mortality following lung cancer (1). According to global cancer statistics from 2020, prostate cancer was the fourth most common cancer worldwide, accounting for 3.7% of new cases diagnosed that year, as estimated across 36 cancers in 185 countries (2). Another study reported approximately 1,276,000 new cases and 359,000 deaths globally in 2018 attributed to prostate cancer (3). Projections indicate a significant rise in the global burden of prostate cancer, with an estimated 3.2 million new cases and 740,000 deaths by 2040, primarily due to population growth and aging demographics (4). A longitudinal study examining prostate cancer incidence in Iran over a 27-year period revealed a rate of 8.24 per 100,000 individuals in 2017, marking a 27.11-fold increase compared to earlier data (5). Prostate cancer is the most frequently diagnosed malignancy among Iranian men, ranking second after stomach cancer (6). The standardized incidence rate of cancer in Iran is reported at 6.11 per 10,000 individuals, with the lowest incidence observed in Kerman at approximately 3.2 per 10,000. This discrepancy may stem from lifestyle factors or a higher prevalence of other diseases (5). Key risk factors for developing prostate cancer include age, race, hereditary predisposition, genetic influences, dietary habits, obesity, prostatitis, hormonal factors, sexual behavior patterns, alcohol consumption, and ultraviolet exposure (1).

The post-anesthesia care unit (PACU), referred to as the "recovery room" in some regions, is designed and equipped to care for patients recovering from anesthesia or surgical procedures (4). This unit is managed by specialized nurses and expert anesthesiologists under the supervision of an anesthesiologist. Familiarity with principles of safe healthcare is essential for recovery personnel (6). The recovery room is a critical area in the hospital, as patients are at high risk of unintentional injury during this time. Patients are often in an unstable physiological state, making them susceptible to rapidly developing critical conditions. Many adverse events are preventable, but their identification and management rely on skilled, vigilant personnel capable of continuous care (7). Complications in intensive care settings include drug reversal effects, upper airway obstruction, loss of pharyngeal muscle tone, residual neuromuscular blockade, laryngospasm, obstructive sleep apnea, pulmonary shunting, cardiac dysrhythmias, delirium, postoperative urinary retention, chills, nausea, and postoperative vomiting (3). Hemodynamic instability—manifested as fluctuations in blood pressure or heart rate—also poses significant risks (8)]. Proper discharge protocols in the PACU are essential for minimizing complications and enhancing safety (9).

Despite advancements in prostate cancer care and the adoption of safety checklists in PACU settings, limited evidence exists comparing the efficacy of SAMPE and ALDERTE checklists in reducing post-surgical complications. This study seeks to address this gap by evaluating these tools in a high-risk population undergoing prostatectomy (9). The transition of patients from the PACU to other hospital departments or home discharge represents a critical phase in ensuring the safe transfer of surgical patients. This process is vital for maintaining physiological stability and preventing adverse effects or errors during transfer (9, 10). Evaluating clinical scenarios of patients with varying postoperative conditions can be complex, often relying on the subjective judgment of healthcare personnel. Recovery continues from the conclusion of intraoperative care until the patient returns to their preoperative physiological state (10). Failure to implement standard strategies for transferring patients between care units can result in harm, increased healthcare costs, and dissatisfaction (11). Health is a fundamental human need and a right for all individuals globally, playing a vital role in sustainable development and achieving numerous social and economic goals. Consequently, patient safety has become integral to healthcare systems worldwide. Clinical errors frequently arise from individual mistakes and systemic weaknesses in healthcare services, emerging as a significant global issue and a key indicator of patient safety (12, 13).

Various anesthesia checklists have been introduced to reduce human error, enhance patient safety, minimize hospitalization durations, improve satisfaction, lower mortality rates, and reduce costs (4). These initiatives facilitate timely patient discharge while mitigating errors and optimizing resource utilization (8). A study by Tevis et al. in 2014 revealed that 42% of complications manifest after discharge. Proper implementation of discharge processes and checklists has improved re-hospitalization rates and reduced post-surgical complications (14, 15). Regulatory associations globally mandate policies to ensure safe postoperative recovery (15, 16). Checklists in clinical settings expedite treatment processes and minimize errors in high-stress environments through optimized preparedness and management. Evidence suggests safety checklists significantly enhance patient safety outcomes (17).

Despite systems assessing readiness for discharge after anesthesia, comparative studies evaluating the impact of these tools on post-discharge complications from the PACU remain limited (18). Effective tools must be functional, user-friendly, and applicable in diverse post-anesthesia scenarios (19). Given the critical importance of reducing complications during patient discharge and the pivotal role of pre-anesthesia assessments, this study underscores their significance in enhancing patient safety.

## 2. Objectives

This research aims to address the critical issue of reducing post-surgical complications by optimizing the discharge process and minimizing human errors. The study hypothesizes that the SAMPE checklist, with its broader inclusion of parameters, would outperform the ALDERTE checklist in managing complications such as bleeding and vomiting, while both would show comparable outcomes in other parameters like pain management. By comparing the ALDERTE and SAMPE checklists and highlighting the importance of pre-anesthesia assessments, the study contributes to a better understanding of how to enhance patient safety during the discharge phase. Additionally, the study endeavors to identify specific strengths and limitations associated with each checklist, providing a foundation for refining postoperative protocols to align with patient safety standards.

## 3. Methods

This descriptive-analytical cross-sectional study aimed to determine the distribution of complications following radical prostatectomy surgery by utilizing two discharge checklists, SAMPE and ALDERTE, from the PACU. Conducted from January to December 2022 across three urban tertiary-care teaching hospitals, the study included 156 patients, divided into three groups of 52 individuals each. The sample size was determined using a power analysis, assuming a medium effect size (Cohen's d = 0.5), a power of 80%, and a significance level of 0.05.

Inclusion criteria encompassed individuals aged 50 to 75 years, classified under ASA physical status 1, 2, or 3, admitted from inpatient wards, possessing awareness of time and place, adequate speech capabilities, and access to mobile phones for postoperative telephone follow-up. Non-inclusion criteria included unwillingness to cooperate, cognitive impairment, and limitations affecting postoperative speech, as these factors precluded participation from the outset. Exclusion criteria included transfer to the post-operative intensive care unit, general anesthesia exceeding three hours, and a history of warfarin use within five days prior to surgery, as these conditions necessitated removal from the study.

The age range of 50 to 75 years was selected as it represents the demographic with the highest incidence of prostate cancer. ASA physical status 1 - 3 was chosen to exclude individuals with severe systemic diseases. Postoperative speech limitations were excluded to ensure accurate self-reporting during follow-up. Subgroup analyses were performed based on age groups (50 - 60 and 61 - 75 years) and ASA status (1, 2, 3). Adjustments for confounders, such as pre-existing comorbidities, were conducted using multivariate logistic regression.

The study adhered strictly to ethical guidelines, including obtaining informed consent from all participants and securing approval from the institutional ethics review board.

## 4. Results

Table 1 summarizes the demographic and lifestyle characteristics of the participants in the study groups. No significant differences were observed among the groups in terms of age, body mass index, activity level, marital status, or surgical history. These findings indicate that the groups were well-matched with respect to key demographic and lifestyle factors, eliminating confounding variables that could potentially skew the comparative analysis of checklist performance.

**Table 1.** Frequency Distribution of Variables in Groups ^a^

Variables	ALDERTE Group (n = 52)	SAMPE Group (n = 52)	Control Group (n = 52)	P-Value
**Age (y)**	64.60 ± 0.935	65.58 ± 0.891	63.58 ± 0.667	0.993
**BMI (average)**	22.68 ± 0.375	22.71 ± 0.387	23.17 ± 0.214	0.588
**ASA class**				0.904
1	6	7	6	
2	40	37	41	
3	6	8	5	
**Activity rate**				0.355
< 30 min	7	8	2	
30 - 60 min	30	29	34	
> 60 min	15	15	16	
**Educational status**				0.237
Illiterate	2	2	0	
Under The diploma	9	15	7	
Diploma	20	14	24	
Academic	21	21	21	
**Marital status**				0.891
Single	0	0	0	
Married	47	47	47	
Divorced	2	3	1	
Widowed	3	2	4	
**Surgery history**				0.795
Yes	45	42	44	
No	7	10	8	

^a^ Values are expressed as mean ± SD or No.

Table 2 presents a comparison of the participants' past medical histories. While no significant differences were found among the groups, hypertension and cancer were identified as common comorbidities, highlighting the need for vigilant postoperative care in managing these conditions.

Table 3 outlines the postoperative complications experienced by the patients. Hematuria, bleeding, and constipation emerged as the most frequent complications. However, no statistically significant differences were observed in the rates of these complications among the three groups, suggesting comparable effectiveness across the applied discharge protocols.

**Table 2.** Frequency Distribution of Past Medical Patients’ History

History	ALDERTE Group (n = 52)	SAMPE Group (n = 52)	Control Group (n = 52)	P-Value
**Diabetes**	17	19	19	0.993
**Hypertension**	23	29	19	0.196
**Cardiac diseases**	12	18	8	0.760
**Cancer**	27	32	34	0.394
**Tobacco**	14	18	11	0.331
**Alcohol**	9	9	8	> 0.05
**Inheritance**	26	31	22	0.233
**Warfarin **	8	16	7	0.063
**Herbal plants**	3	3	1	0.700
**Allergy**	11	15	8	0.273

**Table 3.** Distribution of Complications After Discharge of Patients

Variables	ALDERTE Group (n = 52)	SAMPE Group (n = 52)	Control Group (n = 52)	P-Value
**Nausea**	19	25	17	0.250
**Vomiting**	7	19	4	0.001
**Dizziness**	12	10	21	0.039
**Shivering**	14	14	14	> 0.05
**Pain**	19	18	23	0.625
**Constipation**	47	43	45	0.564
**Shortness of breath**	3	1	1	0.620
**Bradycardia**	2	2	0	0.547
**Tachycardia**	1	4	1	0.368
**Hematuria**	50	51	46	0.149
**Bleeding**	49	48	44	0.314
**Hypotension**	2	6	2	0.221
**Hypertension**	6	6	1	0.104
**Headache**	2	1	0	0.773
**Tachypnoea**	0	0	0	< 0.001
**Rupture of the rectum**	0	0	0	< 0.001
**Blood transfusion after surgery**	12	21	11	0.069
**Blood transfusion during surgery**	15	27	12	0.005

## 5. Discussion

This study aimed to evaluate the comparative efficacy of the SAMPE and ALDERTE checklists in managing complications post-surgery. The key finding was that both checklists performed comparably overall, with minor variations in outcomes such as bleeding and vomiting. These findings highlight the need for further research to optimize checklist criteria. Previous research focused solely on comparing these tools at discharge, neglecting post-discharge complications. This study sought to fill this gap by evaluating the long-term efficacy of these tools.

Given the importance of the topic and the scarcity of investigations concerning radical prostatectomy, this surgery was selected as the focal point of the research. The primary objective was to assess the sensitivity and characteristics of each checklist in identifying early and late postoperative complications in radical prostatectomy patients, ultimately aiming to determine superior performance. Due to the novelty of the SAMPE checklist, there is a lack of studies evaluating its postoperative outcomes. The SAMPE checklist functions as a conservative tool, and a basic but incomplete correlation between SAMPE and ALDERTE was anticipated (19).

El Aoufy et al. highlights that while SAMPE and ALDERTE are valuable tools for managing post-anesthesia recovery, the clinical outcomes and complications associated with their use remain under-researched. The review emphasizes the importance of evaluating postoperative complications after discharge, noting that discharge readiness tools often lack longitudinal follow-up on patients. It stresses the need for discharge readiness checklists to address long-term complications for comprehensive patient management (20).

In the ALDERTE group, 18 complications were assessed, with nausea reported in 12.2% of the sample, closely aligning with the control group at 10% but outperforming the SAMPE group at 16%. This 5.4% difference suggests effective management by the ALDERTE group. Dizziness was reported in 7.7% of samples across both groups, with better outcomes than the control group. Constipation was prevalent, affecting over 80% of the sample.

Two other recurrent conditions, hematoma and wound-site bleeding, were observed in over 90% of samples across all groups. The average bleeding rate was approximately 600 cc, with the SAMPE group showing the highest blood product use. This underscores the need for precise monitoring and individualized care.

The SAMPE checklist group exhibited a higher incidence of vomiting, with twice as many cases as the ALDERTE group, though ALDERTE demonstrated superior management despite not including vomiting in its discharge criteria. Tachycardia was more frequent in the ALDERTE group, though the small sample size limits statistical significance. Tremor control was consistent across all groups. The control group had the highest incidence of post-discharge pain, emphasizing the checklists’ effectiveness in pain management. Among the 18 complications, hematuria, surgical site bleeding, and constipation were the most significant. Notably, the SAMPE group’s increased blood product use raises concerns about its role in bleeding management, suggesting a need to examine whether it delays detection or impacts clinical decisions. These findings may reflect procedural differences in SAMPE implementation or patient variations, necessitating further investigation.

In conclusion, while no overall superiority was observed across the three groups in managing complications, higher bleeding rates in the SAMPE group warrant further study. Addressing these differences could enhance checklist utility and inform strategies to mitigate complications. Gholamzadeh et al. evaluated the psychometric properties of the Persian version of the SAMPE checklist, providing insights into reliability and validity but not addressing complications, highlighting the need for future research. Pain management emerged as a strength of both checklists compared to the control group, underscoring their clinical utility (21).

Ekoff et al. found RDAT effective for discharge readiness but did not explore post-discharge complications, leaving gaps in understanding the tool’s long-term impact (22). Similarly, Street et al. studied discharge criteria tools, noting improvements in nurses' responses and PACU efficiency but not addressing post-discharge complications (23). Overall, these studies provide valuable insights but collectively underscore a significant oversight: The lack of focus on post-discharge complications, which remains an important area for future research.

### 5.1. Conclusions

The present study demonstrated that the two checklists evaluated did not confer a significant advantage over the control group, which adhered to routine discharge protocols at the same treatment center. The performance metrics of both checklists were comparable, with each showing superior outcomes in specific contexts, yet neither emerged as universally superior. Notably, the SAMPE checklist, while incorporating assessments for nausea and vomiting, outperformed the ALDERTE checklist in certain parameters. However, despite the SAMPE group's protocol for monitoring bleeding prior to discharge, the incidence of bleeding and blood transfusion requirements were notably higher in this cohort. These higher bleeding rates raise concerns about its effectiveness in high-risk scenarios, necessitating further refinements.

When assessing other complications, all three groups exhibited similar efficacy, indicating that the choice of checklist should be tailored to the individual patient's condition and the specific treatment center, as determined by the anesthesiologist's discretion. Future randomized controlled trials with larger sample sizes are recommended to validate these findings and establish standardized protocols. This paper encountered no significant limitations, enabling an unrestricted and thorough examination of the research topic, ensuring a robust and detailed discussion.

## Data Availability

The dataset used in this study is available upon request from the corresponding author due to privacy concerns related to patient data.

## References

[1] Marashi T, Razaghi M, Khodakarim S, Balvayeh M (2017). [Surveying the Awareness of Male Hospital Staffs about Prostate Cancer Screening in Selected Hospitals of Shahid Beheshti University of Medical Sciences in 2017].. Paramedic Sci Milit Health..

[2] Sung H, Ferlay J, Siegel RL, Laversanne M, Soerjomataram I, Jemal A (2021). Global Cancer Statistics 2020: GLOBOCAN Estimates of Incidence and Mortality Worldwide for 36 Cancers in 185 Countries.. CA Cancer J Clin..

[3] Basiri A, de la Rosette JJ, Tabatabaei S, Woo HH, Laguna MP, Shemshaki H (2018). Comparison of retropubic, laparoscopic and robotic radical prostatectomy: who is the winner?. World J Urol..

[4] Deshmukh PP, Chakole V (2024). Post-Anesthesia Recovery: A Comprehensive Review of Sampe, Modified Aldrete, and White Scoring Systems.. Cureus J..

[5] Momayyezi M, Dehghani Tafti A, Keyghobadi N, Fallahzadeh H, Mirzaei M (2022). Prevalence of Prostate Cancer and its Risk Factors: Results of the First Phase of Shahedieh Cohort Study in Yazd Province, Iran.. J Prevent Med..

[6] Sarbaz Aqdaee F, Derhami V, Zare Sakhvidi MJ, Mostaghaci M, Soltani Gerdfaramarzi R, Musavi M (2016). [Diagnosis of prostate cancer and predicting the probability of suffering the disease in workers].. Occup Med Qtly J..

[7] Talebi Moghaddam M, Bakhshi E, Amini E, Nowroozi MR, Vahedi M (2021). Effects of Admission Age and Gleason Score on the State Transition in Elderly Patients With Prostate Cancer Using a Multi-State Model.. Salmand J..

[8] Hatfield A (2014). The Complete Recovery Room Book (5 ed)..

[9] Nazemroaya B, Keleidari B, Arabzadeh A, Honarmand A (2022). Comparison of Intraperitoneal Versus Intravenous Dexamethasone on Postoperative Pain, Nausea, and Vomiting After Laparoscopic Cholecystectomy.. Anesth Pain Med..

[10] Advani R, Stobbs NM, Killick N, Kumar BN (2015). “Safe handover saves lives”: results from clinical audit.. Clinic Governance: An Int J..

[11] Marshall SI, Chung F (1999). Discharge criteria and complications after ambulatory surgery.. Anesth Analg..

[12] Prates A, Colognese B, Caumo W, Stefani LC (2022). Development of a recovery-room discharge checklist (SAMPE checklist) for safe handover and its comparison with Aldrete and White scoring systems.. Braz J Anesthesiol..

[13] Baker GR, Norton PG, Flintoft V, Blais R, Brown A, Cox J (2004). The Canadian Adverse Events Study: the incidence of adverse events among hospital patients in Canada.. Canadian Med Assoc J..

[14] Friedenthal J, Maxwell SM, Tiegs AW, Besser AG, McCaffrey C, Munne S (2020). Clinical error rates of next generation sequencing and array comparative genomic hybridization with single thawed euploid embryo transfer.. Eur J Med Genet..

[15] Tevis SE, Kohlnhofer BM, Weber SM, Kennedy GD (2014). Postdischarge complications are an important predictor of postoperative readmissions.. Am J Surg..

[16] Merchant R, Chartrand D, Dain S, Dobson G, Kurrek MM, Lagacé A (2015). Guidelines to the Practice of Anesthesia – Revised Edition 2016.. Canadian J Anesth..

[17] Scott AM, Li J, Oyewole-Eletu S, Nguyen HQ, Gass B, Hirschman KB (2017). Understanding Facilitators and Barriers to Care Transitions: Insights from Project ACHIEVE Site Visits.. Jt Comm J Qual Patient Saf..

[18] Obstetric Anaesthetists' Association (2024). Checklist..

[19] Awad IT, Chung F (2006). Factors affecting recovery and discharge following ambulatory surgery.. Can J Anaesth..

[20] El Aoufy K, Forciniti C, Longobucco Y, Lucchini A, Mangli I, Magi CE (2024). A Comparison among Score Systems for Discharging Patients from Recovery Rooms: A Narrative Review.. Nurs Rep..

[21] Gholamzadeh M, Saei A, Sedigh Maroufi S, Khobbin Khoshnazar TA, Rajabzadeh R (2023). The Psychometry and Localization of the Patient Assessment and Discharge Checklist in the Post-Anesthesia Care Unit (SAMPE Checklist).. J Police Med..

[22] Ecoff L, Palomo J, Stichler JF (2017). Design and Testing of a Postanesthesia Care Unit Readiness for Discharge Assessment Tool.. J Perianesth Nurs..

[23] Street M, Phillips NM, Kent B, Colgan S, Mohebbi M (2015). Minimising post-operative risk using a Post-Anaesthetic Care Tool (PACT): protocol for a prospective observational study and cost-effectiveness analysis.. BMJ Open..

